# Reconstruction of Electrical Burn Wounds Using Acellular Dermal Matrix: A Case Report and Literature Review

**DOI:** 10.7759/cureus.90071

**Published:** 2025-08-14

**Authors:** Rodrigo Davila-diaz, Edwing Michel Jaimes-Duran, Mauricio Gutierrez-Alvarez, Carlos Cortes-Aguilar, Luis A Camarillo Reyes, Mely Alondra Mendizabal Velazquez, Gabriela Alejandra Wong Ruiz, Cuahutemoc Marquez-Espriella

**Affiliations:** 1 Plastic and Reconstructive Surgery, Hospital Central Sur de Alta Especialidad Petróleos Mexicanos (PEMEX), Mexico City, MEX; 2 General Surgery, Hospital Central Sur de Alta Especialidad Petróleos Mexicanos (PEMEX), Mexico City, MEX

**Keywords:** acellular dermal matrix, complex wound, electric burn, skin graft, tissue engineering

## Abstract

Electrical burns present a therapeutic challenge due to the depth and extent of tissue damage. Acellular dermal matrix (ADM) has emerged as a promising alternative for complex wound coverage. Our objective is to describe the use of ADM in electrical burns. We present the case of a 52-year-old male patient with an 11.5% total body surface area electrical burn on the left side of the neck who was treated with surgical debridement and ADM application directly onto the prepared wound bed and secured with sutures and dressings. It acts as a scaffold for the patient's tissue regeneration, and an autologous skin graft is subsequently placed. Tissue integration and functional outcomes were evaluated six months after the initial injury. ADM facilitated dermal regeneration, reduced skin contracture, and improved both cosmetic and functional outcomes. ADM is a viable option for the management and coverage of electrical burn injuries, promoting wound healing and reducing complications.

## Introduction

Electrical burns represent a challenge for healthcare professionals and plastic surgeons due to their unique mechanism of injury, which combines thermal trauma, electrical current damage, and deep tissue involvement [1]. Unlike conventional thermal burns, electrical injuries follow an “iceberg effect” pattern, where superficial damage may underestimate extensive necrosis in deeper structures such as muscle, nerves, and blood vessels [2]. Due to this characteristic, surgical management with sequential debridement will be needed, leading to complex tissue defects that will require advanced reconstructive strategies [3].

Traditionally, wound closure has relied on autologous skin grafts, local, or free tissue flaps. However, in cases involving extensive tissue loss, the limited availability of donor sites poses a major limitation [4]. Furthermore, thin skin grafts applied directly over nonviable tissue or exposed structures (such as tendons or bone) are associated with high failure rates [5].

In this context, acellular dermal matrix (ADM) has emerged as a promising reconstructive option. These dermal substitutes, derived from decellularized human or porcine tissue, provide a biocompatible scaffold that facilitates cellular infiltration, neovascularization, and integration with host tissue [6]. While several studies have demonstrated their efficacy in thermal burns, their application in electrical injuries remains less well documented [7]. This article presents a successful case of ADM (Integra®, Integra LifeSciences Corporation, Plainsboro, NJ), which is an advanced biomaterial used in reconstructive surgery and tissue regeneration. This product is designed to temporarily replace lost or damaged dermis, providing a three-dimensional structure that supports the patient’s tissue regeneration. ADMs were used in a patient with a severe electrical burn, and reviews current evidence regarding their benefits, limitations, and application protocols.

## Case presentation

**Figure 1:**
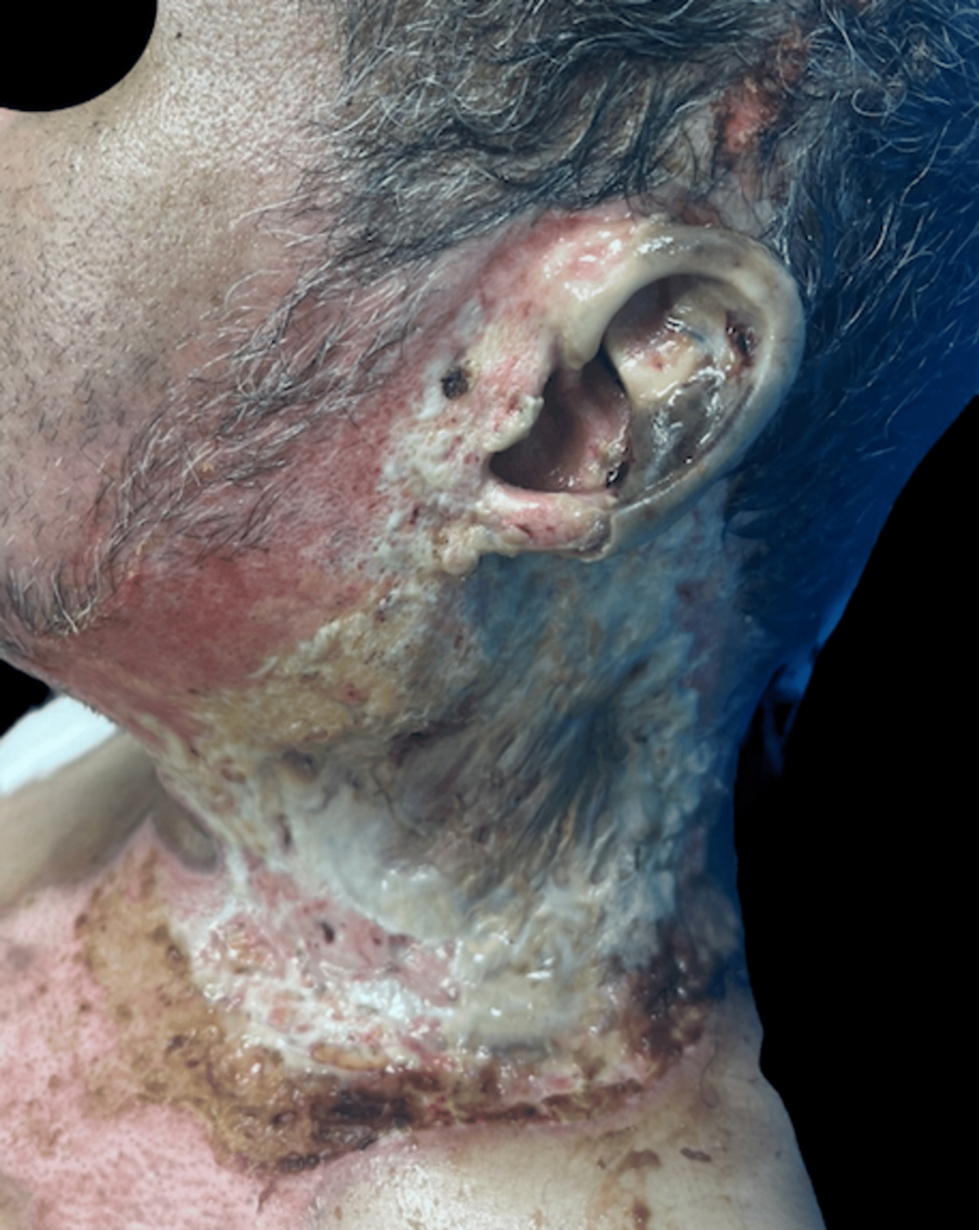
Photograph at the time of patient admission Photograph showing the patient with a burn on the neck. The lesion exhibits devitalized, leathery tissue with involvement of the auricular framework. There is a noticeable limitation of neck movement. Erythema is present at the periphery of the lesion, with no signs of infection. Fibrinous tissue is observed within the wound bed.

The patient was A 52-year-old male with no significant past medical history. The patient sustained a high-voltage electrical injury due to direct contact with overhead power lines during home maintenance activities. This resulted in electrical burns complicated by secondary ignition of clothing, causing extensive full-thickness (third-degree) thermal injuries involving the cervical region (Figure 1), thorax, and upper extremities. The total body surface area affected was estimated at 11.5%. The patient was managed according to established advanced burn care protocols; this management of patients with high-voltage electrical burns follows the structured trauma approach, beginning with the Airway, Breathing, Circulation, Disability, Exposure (ABCDE) primary assessment. Airway patency is ensured early, especially in cases with suspected inhalation injury or facial burns. Breathing is evaluated for signs of respiratory compromise. Circulation is assessed with early initiation of cardiac monitoring due to the high risk of arrhythmias, including ventricular fibrillation or asystole. Disability assessment includes a neurological evaluation, as electrical injuries can cause immediate central or peripheral nervous system dysfunction.

Aggressive fluid resuscitation was initiated based on the Parkland formula, with adjustments to maintain adequate urine output (≥1 mL/kg/h). In the presence of rhabdomyolysis, the urine output target is increased to 1.5-2 mL/kg/h, and urinary alkalinization with sodium bicarbonate may be considered. Continuous ECG monitoring for at least 24 hours is essential, along with frequent laboratory evaluations, including serum electrolytes, creatine phosphokinase (CPK), and serum/urinary myoglobin, to monitor for renal impairment and muscle damage.

Close surveillance for early signs of compartment syndrome is mandatory, and fasciotomies are performed promptly if indicated to preserve limb viability. Given the potential for associated head and neck injuries, consultations with otolaryngology and ophthalmology are obtained to evaluate for trauma to the face, globe, middle ear, or inner ear structures.

This comprehensive, multidisciplinary approach allows early detection and management of life-threatening complications and optimizes patient outcomes in high-voltage electrical injuries.

**Figure 2:**
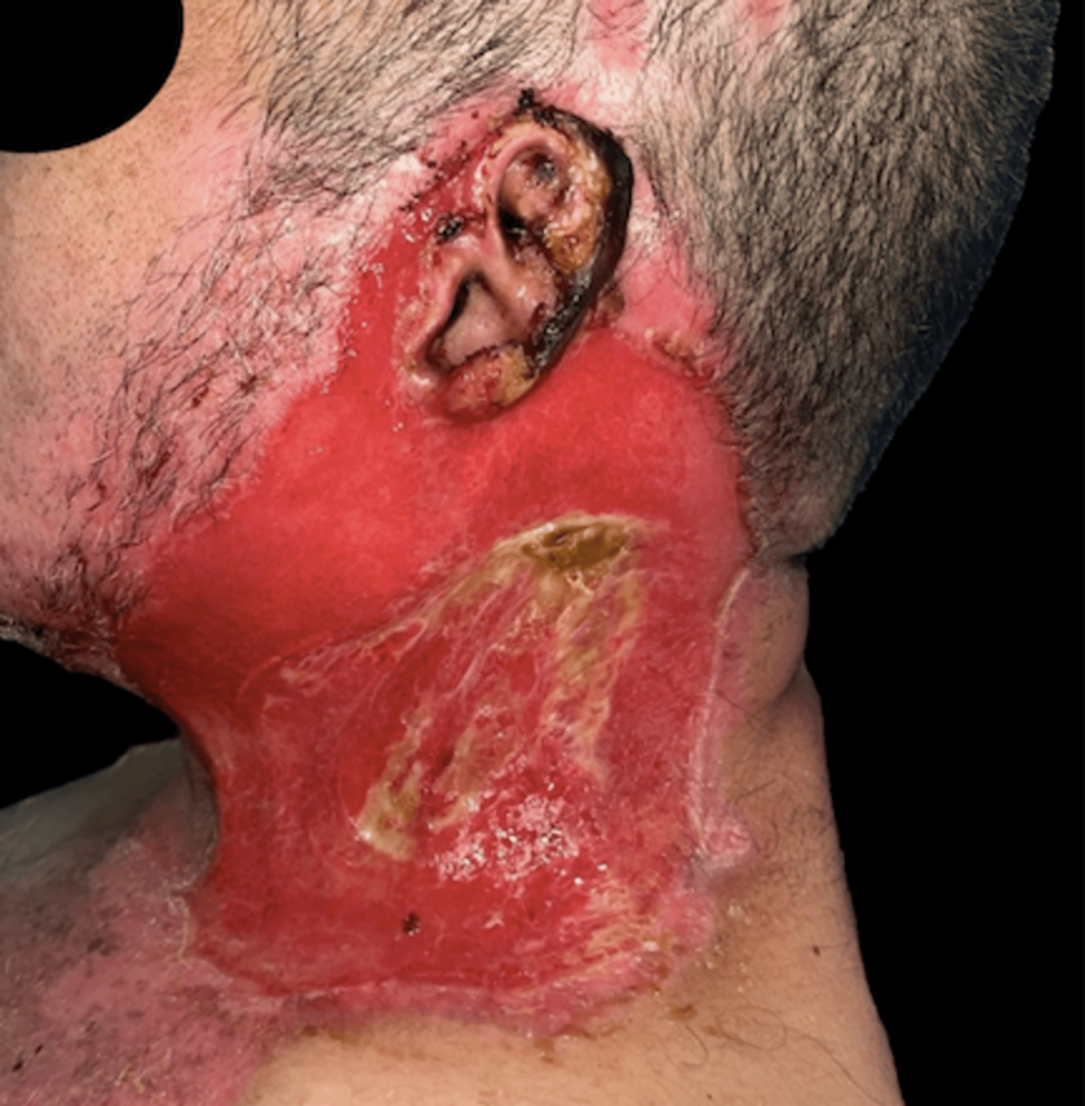
Patient after mechanical wound cleansing and eschar excision of the left side of the neck. A lesion with underlying erythematous tissue beneath the eschar is seen. Previously devitalized tissue has been debrided, with small areas of fibrin persisting in the center. No signs of infection are observed.

Initial treatment involved mechanical wound cleansing using antiseptic solutions (Microdacyn®) and cervical eschar excision (Figure 2). Surgical management proceeded with serial debridements, continuing until viable, non-necrotic tissue was identified. Between debridement sessions (a total of three), topical ointments (Ulcoderma®) containing chloramphenicol and collagenase were applied to promote enzymatic debridement, all under close monitoring of systemic response.

The patient developed rhabdomyolysis secondary to deep muscle injury, which was managed with aggressive fluid resuscitation and metabolic surveillance. As previously mentioned, urine output targets were increased to 1.5-2 mL/kg/h to prevent renal complications associated with myoglobinuria.

**Figure 3:**
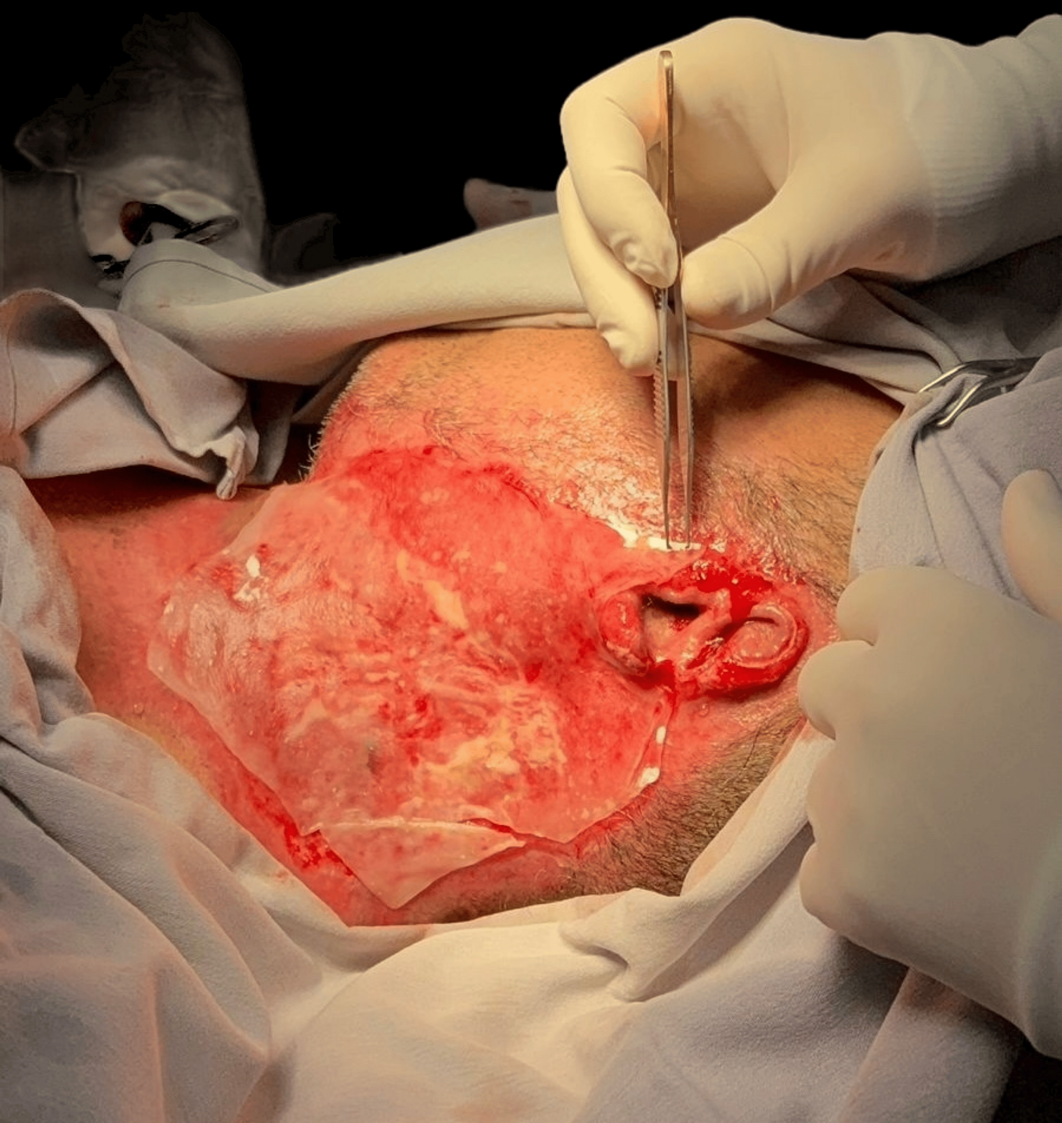
Acellular dermal matrix being placed on the cervical region.

**Figure 4:**
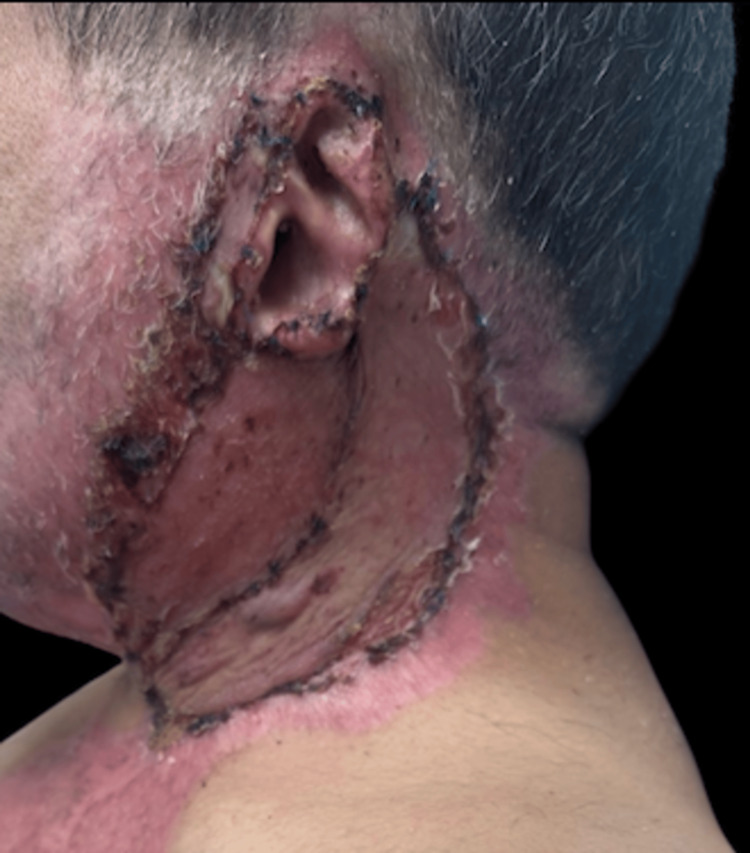
Seven-day follow-up showing complete integration of the full-thickness skin graft.

An ADM was applied to the cervical region to promote tissue integration and reduce the risk of scar contracture (Figure 3). A split-thickness skin graft (STSG) harvested from the anterolateral region of the right thigh was performed seven days later, with adequate wound coverage (Figure 4).

Functional preservation of the affected areas was achieved. No functional limitations or evidence of secondary contracture were observed during follow-up six months after the initial injury. This was evaluated through physical examination, where the patient demonstrated adequate cervical range of motion, including flexion, extension, and rotation, with all movement arcs above 90%, without restrictions. In our case, there were no signs of infection, and the graft exhibited characteristics similar to the adjacent skin, with no evidence of pathological scarring or secondary contracture.

In contrast, many patients treated without the use of dermal matrix often present with movement limitations that impair daily activities and reduce quality of life, increasing morbidity and mortality. Such patients frequently require subsequent surgical procedures, including en bloc scar resections and Z-plasty, among others.

The timely and multidisciplinary approach resulted in a favorable clinical outcome in the context of a high-voltage electrical burn. The advantages of this strategy include reduced morbidity and mortality, preservation of internal organ function (renal), limitation of tissue damage and burn depth by preventing the progression of Jackson’s burn zones, and improved functional outcomes with unrestricted range of motion due to minimized secondary contracture formation. Long-term prognosis remains guarded and will depend on the patient's functional recovery, emotional adjustment, and response to a comprehensive rehabilitation program. Follow-up will be provided for at least one year after the procedure.

## Discussion

ADM can be used as a temporary coverage strategy prior to autologous skin grafting in the management of burn injuries. In this case, the tissue engineering approach involved an acellular artificial dermis composed of bovine collagen and glycosaminoglycans, overlaid with a silicone layer designed to mimic the function of the epidermis [8]. Its application following the initial surgical debridement served three critical purposes.

The silicone layer minimized fluid loss and reduced the risk of bacterial contamination, key factors in patients with prolonged exposure of critical structures [9]. Comparative studies have reported a 40% reduction in infection rates when ADM is used instead of conventional dressings in complex wounds [10].

Guided dermal regeneration and fibroblast and vascular infiltration into the matrix were observed within two weeks, creating a well-vascularized wound bed suitable for subsequent autologous grafting. This correlated with findings from case series reporting skin graft integration rates exceeding 85% when applied over fully integrated ADM [11].

For functional preservation purposes, two months post treatment, the patient demonstrated nearly 90% of the contralateral joint range of motion, with no significant contractures. This contrasts with the typical course of electrical burns treated with direct grafting, where contractures affect up to 60% of cases. ADM appears to mitigate this issue by promoting more organized wound healing, as suggested by a prospective study reporting decreased fibrosis on histological examination in ADM-treated wounds [12].

Despite its advantages, the use of ADM in electrical burn management presents certain limitations, the most notable being its cost. The price per square centimeter of ADM is approximately three to five times higher than that of conventional skin grafts. However, cost-effectiveness analyses indicate it may be economically justified by reducing the need for reoperations [13]. A recent meta-analysis demonstrated that although re-epithelialization may be slightly delayed by approximately five days in patients treated with ADM compared to those treated with STSG alone, scar quality at six months, as assessed by the Vancouver Scar Scale (VSS), was significantly better in the ADM group (p < 0.01). These findings suggest that scars in the ADM group more closely resemble normal skin, exhibiting improved vascularity, pigmentation, texture, pliability, and reduced height [14], similar to what we found in our case.

Delayed integration time and the requirement for a second surgical stage (autologous grafting) typically extend hospitalization by two to four weeks. Finally, the risk of infection and ADM failure has been reported in retrospective series to range from 10% to 15%, frequently associated with colonization by *Pseudomonas *or resistant strains of *Staphylococcus *[15].

Next-generation ADMs combined with adjunctive therapies, such as negative pressure wound therapy or growth factor incorporation, are under investigation. A recent clinical trial showed that ADM impregnated with platelet-derived growth factor (PDGF) reduced integration time by approximately 30% [16]. Additionally, experimental models using "bioactive" matrices capable of releasing antibiotics suggest potential for lowering infectious complications [17]. Future multicenter studies with larger patient cohorts and long-term follow-up are needed to consolidate the role of dermal matrices in standardized protocols for the management of complex electrical burns.

## Conclusions

The management of deep electrical burns remains a significant challenge due to the complexity of tissue damage, exposure of critical structures, and the high risk of functional and aesthetic complications. In this context, the use of ADM has proven to be a valuable tool. The use of ADM provides a biocompatible scaffold that promotes organized dermal regeneration, allowing for safe and effective wound coverage, particularly in areas where direct skin grafting is not feasible due to exposed tendons, bone, or joints, or critical areas. Our results are consistent with those reported in the literature, demonstrating fewer complications, reduced secondary contracture, improved range of motion, and better scar quality while preserving the functional integrity of the recipient site. This two-stage approach of initial placement of the ADM followed by autologous skin grafting has been associated with improved outcomes regarding scar quality, reduction of contractures, and enhanced long-term functionality.
